# The Genetic Structure and East-West Population Admixture in Northwest China Inferred From Genome-Wide Array Genotyping

**DOI:** 10.3389/fgene.2021.795570

**Published:** 2021-12-21

**Authors:** Bin Ma, Jinwen Chen, Xiaomin Yang, Jingya Bai, Siwei Ouyang, Xiaodan Mo, Wangsheng Chen, Chuan-Chao Wang, Xiangjun Hai

**Affiliations:** ^1^ Key Laboratory of Environmental Ecology and Population Health in Northwest Minority Areas, Northwest Minzu University, Lanzhou, China; ^2^ State Key Laboratory of Cellular Stress Biology, School of Life Sciences, Xiamen University, Xiamen, China; ^3^ Department of Anthropology and Ethnology, School of Sociology and Anthropology, Institute of Anthropology, National Institute for Data Science in Health and Medicine, Xiamen University, Xiamen, China; ^4^ State Key Laboratory of Marine Environmental Science, Xiamen University, Xiamen, China

**Keywords:** genetic structure, admixture history, gene flow, west Eurasia, steppe population, trans-Eurasia, gansu, northwest China

## Abstract

Northwest China is a contacting region for East and West Eurasia and an important center for investigating the migration and admixture history of human populations. However, the comprehensive genetic structure and admixture history of the Altaic speaking populations and Hui group in Northwest China were still not fully characterized due to insufficient sampling and the lack of genome-wide data. Thus, We genotyped genome-wide SNPs for 140 individuals from five Chinese Mongolic, Turkic speaking groups including Dongxiang, Bonan, Yugur, and Salar, as well as the Hui group. Analysis based on allele-sharing and haplotype-sharing were used to elucidate the population history of Northwest Chinese populations, including PCA, ADMIXTURE, pairwise Fst genetic distance, *f*-statistics, qpWave/qpAdm and ALDER, fineSTRUCTURE and GLOBETROTTER. We observed Dongxiang, Bonan, Yugur, Salar, and Hui people were admixed populations deriving ancestry from both East and West Eurasians, with the proportions of West Eurasian related contributions ranging from 9 to 15%. The genetic admixture was probably driven by male-biased migration- showing a higher frequency of West Eurasian related Y chromosomal lineages than that of mtDNA detected in Northwest China. ALDER-based admixture and haplotype-based GLOBETROTTER showed this observed West Eurasian admixture signal was introduced into East Eurasia approximately 700 ∼1,000 years ago. Generally, our findings provided supporting evidence that the flourish transcontinental communication between East and West Eurasia played a vital role in the genetic formation of northwest Chinese populations.

## Introduction

The human history of East Asia can be traced back to the Late Paleolithic Age. The anatomically modern humans permanently made an occupation in East Asia about 50,000 years ago (Alexander et al., 2009). Numerous evidences from ancient and present-day human genomes suggested an initial settlement in East Asia about 60,000 years ago and multiple waves of population expansion in Paleolithic and Neolithic periods (Fu et al., 2013; Bai et al., 2020; Zhang et al., 2020). The Pan-Asia project suggested the main southern migration route contributed much more to the peopling of the East Asia compared to the northern migration route by analyzing genome-wide data of 1900 individuals from 73 populations (HUGO Pan-Asian SNP Consortium et al., 2009; Cao et al., 2020). However, paternal Y chromosome and maternal mitochondrial DNA indicated that the gene flows from the west and northern Eurasia into East Asia were through the northern migration route (Su et al., 1999; Wen et al., 2004). East Asia is an important earliest center of animal and plant domestication in the world (Wang et al., 2021a). Paleogenomic studies documented that the genetic diversity in prehistoric Asia was higher than in more recent periods of human history and population migration between northern and southern East Asia that started in Late Neolithic Age influenced the genetic formation of modern East Asiana (Ning et al., 2020; Yang et al., 2020; Wang et al., 2021a; Wang et al., 2021b; Xiaowei et al., 2021). These expansion events were associated with the spread of the major language families existing in East Asia. There is also a remarkable diversity of human languages spoken in East Asia, including Sino-Tibetan, Hmong-Mien, Austroasiatic, Tai-Kadai, Austronesian, Indo-European, Turkic, Mongolic, Tungusic, Japonic, Koreanic, Yukaghiric, and Chukotko-Kamchatkan (Wang et al., 2021a; Uesugi et al., 2021). The formation of East Asians is suggested to has involved genetic contributions from various ancestral human populations (Duan et al., 2018; Sun et al., 2019; Wang et al., 2021a).

The Eastern Steppe is characterized with grasslands, forest steppe, and desert steppe, connecting Russia, Mongolia, and China. The Eastern Eurasian Steppe is home to historic empires of nomadic pastoralists, including Xiongnu, Turkic Khaganate, and the Mongols. The East Steppe have also served as the important communication node between West and East Eurasia. The Central/East Steppe has witnessed intensive East and West communications and interactions in many aspects (Elfari et al., 2005; Hyten et al., 2010; Stoneking and Delfin, 2010; Liu et al., 2018; Chen et al., 2019; Lan et al., 2019; Cao et al., 2020; Tangkanchanapas et al., 2020; Rodin et al., 2021). Historical and archeological studies demonstrated that the western Eurasian cultural factors were once brought into the north region of China through the East-West communication corridors (Sanchez-Burks et al., 2003; Xu, 2008; Ning et al., 2019). In the past, the ancient Silk Road was an important connection of the West Eurasia and China, which contributed much to the intensified transcontinental culture and population communications s between the East and West Eurasia (Cheng, 1985; Robino et al., 2014). The Silk Road was at its most bustling time in Tang Dynasty, but before that time the east-west communication was established for a long time, which could be traced back to the Early Bronze Age (Haak et al., 2015; Goldberg et al., 2017; Lazaridis and Reich, 2017; Saag et al., 2017). The corresponding trans-continental population migration during the Late Neolithic Age, the Bronze Age to the Iron Age and historical period had been demonstrated in the core regions of Siberia (Abelson, 1978; Matsumoto et al., 1995; Hemphill and Mallory, 2004; Maramovich et al., 2008; Jeong et al., 2018; Juras et al., 2020; Stoof-Leichsenring et al., 2020). The archeological evidence supported the interaction between the westward spread of millet agriculture and also the eastward spread of barley and wheat agriculture with population migration (Zohary and Hopf, 1973; Medjugorac et al., 1994; Hemphill and Mallory, 2004; Saisho and Purugganan, 2007; Wang et al., 2016; De Barros Damgaard et al., 2018b; Bento et al., 2018; Jeong et al., 2018). The Trans-Eurasian cultural and genetic exchanges have significantly influenced the demographic dynamics of Eurasian populations (Peel and Talley, 1996; Khan et al., 2017; Miller et al., 2017; De Barros Damgaard et al., 2018a; De Barros Damgaard et al., 2018b; Damgaard et al., 2018; Antwerpen et al., 2019; Coulehan, 2020; Saint Onge and Brooks, 2020; Zhou et al., 2020). The EasternEurasian Forest steppe zone was genetically structured during the Pre-Bronze and Early Bronze Age, with a strong west-east admixture cline of ancestry stretching from Botai in central Kazakhstan to Lake Baikal in southern Siberia, and to the Devil’s Gate Cave in the Russian Far East (Jeong et al., 2020). During the Bronze Age, the eastward migration of Western Eurasian nomadic populations related to Afanasievo and Andronovo Culture into Eastern Steppe have not only influenced the gene pool of eastern Eurasian populations (Ning et al., 2019; Wang et al., 2021a), but also drastically changed lifeways and subsistence on the Eastern Steppe. The milk consumption in Mongolia started prior to 2500 BCE by groups related to Afanasievo and Chemurchek culture (Jeong et al., 2018). Until the Iron Age, the pastoralists established the nomadic empire in Eastern Steppe. The Xiongnu empire was the first historically recorded nomadic empire in Eastern Steppe, which had a profound influence on the demographics and geopolitics of Eurasia by expanding into northern China, southern Siberia, and Central Asia, even as far as the West Eurasian (Damgaard et al., 2018). During 13th century, the Mongols group eventually controlled a vast territory and numerous trade routes stretching from China to the Mediterranean (Jeong et al., 2020). The archaeological evidence showed Mongolia Plateau is a conduit for cultural exchanges between the East and the West Eurasia (Malyarchuk et al., 2016; Wang et al., 2021a; Liu et al., 2021).

Northwest China locates in the west-east Eurasian interaction core region, populations in this region mainly belongs to Altaic language family which includes Mongolic, Turkic, and Tungusic language based on language classification. Modern populations in Northwest China were typical admixtures between populations all around the trans-Eurasia continent (Feng et al., 2017; Yao et al., 2021). Uyghur derived western related ancestry from West Eurasians and South Asians, while the eastern related components were from the East Asians, and the Siberians (Ma et al., 2014; Feng et al., 2017; Heizhati et al., 2020). Gansu province connecting the Hexi Corridor and the Tibetan-Yi Corridor in northwest China is not only takes part in the west-east Eurasian communication, but also plays an important role in the southwards population expansion which contributed to the formation of Tibeto-Burman speaking population (Feng et al., 2020; Luo et al., 2020). Human population genetic researches had been carried out based on low-density genetic markers and limited sample sizes to explore the genetic history of Gansu province (Yao et al., 2016; Yao et al., 2017; Wen et al., 2019). But a comprehensive survey of the genetic diversity and fine-scale genetic structure of Gansu province based on genome-wide data were still sparse. Therefore, to shed more light on the genetic profile of northwest China, 140 individuals from Gansu including Hui, Dongxiang, Bonan, Yugur, and Salar ethnic groups were collected and genotyping with Illumina gene arrays at approximately 700,000 genome-wide single-nucleotide polymorphisms (SNPs). We merged the genotyping data with reference data of worldwide populations, and carried out population genetics analysis to explore the genetic structure and uncovered the admixture history of Altaic speaking populations in Northwest China.

## Materials and Methods

### Ethics Statement

The procedures of the sample collection and the investigations were reviewed and approved by the Medical Ethics Committee of Xiamen University and Northwest Minzu University and were in accordance with the recommendations provided by the revised Helsinki Declaration of 2000. Moreover, our study stuff had already informed these potential participants about our purposes of this project, and every participant in our study had provided the informed consent.

### Sample Collection

**FIGURE 1:**
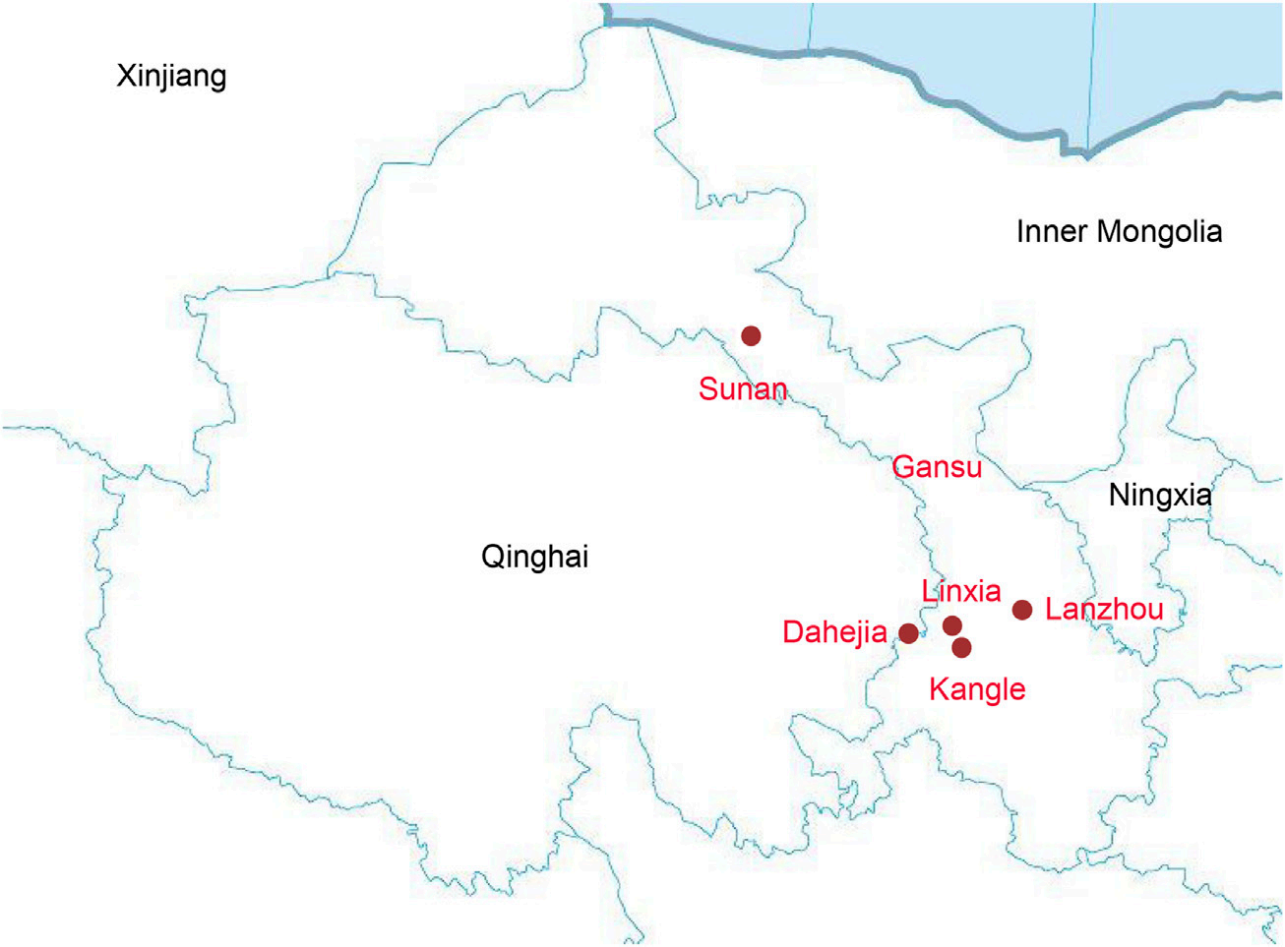
The geographical map of our samples collection.

Our study focused on Gansu province in Northwest China. We collected 140 saliva samples from unrelated individuals of Altaic speaking populations and Hui group from Sunan, Linxia, Lanzhou, Dahejia, and Kangle, including 24 samples from Hui, 30 samples from Dongxiang, 30 samples from Bonan, 30 samples from Yugur, and 26 samples from Salar (Figure 1). All inclued individuals were required to be indigenous self-declared, following the critera that requiring an indigenous person with at least three generations of history in the area and the offspring of a non-consanguineous marriage within populations.s.

### Genotyping and Data Mergeing

We used PureLink Genomic DNA Mini Kit (Thermo Fisher Scientific) to extract DNA and measure the concentration via the Nanodrop-2000 following the manufacturer’s instructions. All these qualified samples were genotyped using the Illumina WeGene Arrays covering about 700,000 Single nucleotide polymorphisms (SNPs) at the WeGene genotyping centre in Shenzhen. We first analyzed the biological relatedness of individuals using plink (Chang et al., 2015) softwere and all individuals were filtered. We also conducted the quality control process. There were 25,653 SNPs which were removed due to high percentage of missingness with “--geno 0.1 –mind 0.1” option using plink. Then we applied a HWE threshold by 0.001, and 17,153 SNPs were removed. We pruned the Linkage Disequilibrium by “--indep-pairwise 200 25 0.4” for ADMIXTURE analysis. We obtained a dataset covring 72,541 SNPs when merged our 140 samples with the previously published data from Human Origin Datasetand a dataset covering merged 95,675 SNPs when merged with 1240 K capture dataset from David Reich Lab (https://reich.hms.harvard.edu/downloadablegenotypes-present-day-and-ancient-dna-data-compiled-published-papers) (Patterson et al., 2006; 2012).

### Principal Component Analysis

Principal component analysis (PCA) was carried out using the software called *smartpca* built in the EIGENSOFT package (Patterson et al., 2006). The PCA analysis was performed at the individual level to describe the genetic structure of all of our samples in Gansu province and the reference populations. We used the following parameters: the numoutlieriter: 0 and lsqproject: YES options. We projected ancient individuals onto the first two components calculated by present-day samples. We visualized the PCA results by the ggplot2 package in the R software (http://www.r-project.org/).

### ADMIXTURE

We carried out ADMIXTURE (Alexander et al., 2009) analysis after pruning for strong linkage disequilibrium in Plink V.1.9 (Purcell et al., 2007; Chang et al., 2015) with the parameters “-indep-pairwise 200 25 0.4”. We ran ADMIXTURE with the 10-fold cross-validation (−CV = 10), varying the number of ancestral populations between K = 2 and K = 20 in 100 bootstraps with different random seeds. We chose the best run according to the highest log-likelihood with the lowest CV error value.

### *F*-Statistics

We computed *f* statistics using ADMIXTOOLS with the default parameters, and calculated standard errors (statistical significance) using a block jackknife resampling across the genome (Patterson et al., 2006). We carried out outgroup *f*_*3*_-statistics of the form *f*_*3*_ (X, Y; Mbuti) to measure the shared genetic drifts between population X and population Y since their separation from an outgroup population. We here used Mbuti as an outgroup population, a group who lived in the Congo basin in the middle region of Africa. We next used admixture-*f*_*3*_ statistics in the form of *f*_*3*_ (X, Y; Target) for all pairs of references populations to make an evaluation of the possible admixture signals for the target populations. We conducted the heatmap visualization of the outgroup-*f*_*3*_ statistics values by the pheatmap package in the R software.

### Streams of Ancestry and the Inference of Admixture Proportions

We investigated the admixture source numbers, plausible admixture sources, and the corresponding admixture proportions based on *qpAdm* program as implemented in ADMIXTOOLS (Patterson et al., 2006). We used this *f*_*4*_-statistics based admixture modeling to explore whether a batch of target populations were consistent with being related via N streams of source populations from a basic set of some outgroups and calculated the admixture proportions of the given source populations quantitatively.

### Y-Chromosomal and mtDNA Haplogroup Assignment

We assigned the Y chromosomal haplogroups by genotyping the most derived allele upstream and the most ancestral allele downstream in the phylogenetic tree by using an in-house script following the recommendations of the International Society of Genetic Genealogy (ISOGG; http://www.isogg.org/). The mtDNA haplogroups assignment was identified with mtDNA phylogenetic tree Build 16 (http://www.phylotree.org/).

### Fst Calculation

The Fst values were calculated by the *smartpca* of EIGENSOFT (Patterson et al., 2006). We ran the *smartpca* with the parameters: inbreed: YES and fstonly: YES, and then output the results by phylipoutname parameter. We found that the inbreeding corrected and uncorrected Fst were nearly identical. In the following, we performed the phylogenetic tree by the Fst values of the populations in Eurasia. We performed the phylogenetic tree by the NJ tree using MEGA software (Kumar et al., 2016).

### Weighted Linkage Disequilibrium Analysis

Linkage disequilibrium decay was computed by ALDER (Loh et al., 2013) to infer the admixture time for our studied populations.

### Fine-Scale Genetic Structure Based on FineSTRUCTURE

Bayesian clustering implemented in FineSTRUCTURE was used to reconstruct polygenetic relationships and further identify population structure. To reduce the computational burden, we selected 10–20 individuals randomly in a reference group and 15 individuals in our studied group. We phased genome-wide dense SNP data using the SHAPEIT2 (Delaneau et al., 2013) and then conducted FineSTRUCTURE (Lawson et al., 2012) analysis.

### ChromoPaintev2 and GLOBETROTTER Admixture Modeling

We performed a GLOBETROTTER (Hellenthal et al., 2014) analysis for our studied groups to obtain haplotype-sharing based evidence of admixture. Using these haplotypes from SHAPEIT2, the “chunk length” output was obtained by running ChromoPainterv2 across all chromosomes. We ran GLOBETROTTER to estimate admixture events by 100 bootstrap replicates, assuming that there is detectable admixture using the “pro.ind:1”, and “bootstrap.date.ind:1” options.

### Runs of Homozygosity

We calculated the Runs of homozygosity by PLINK software. The related parameters were: “--homozyg-density 50, --homozyg-window-het 1, --homozyg-window-threshold 0.05”. Then we presented the counts and lengths of ROH.

## Results

### Population Genetic Structure of the Northwest China

**FIGURE 2:**
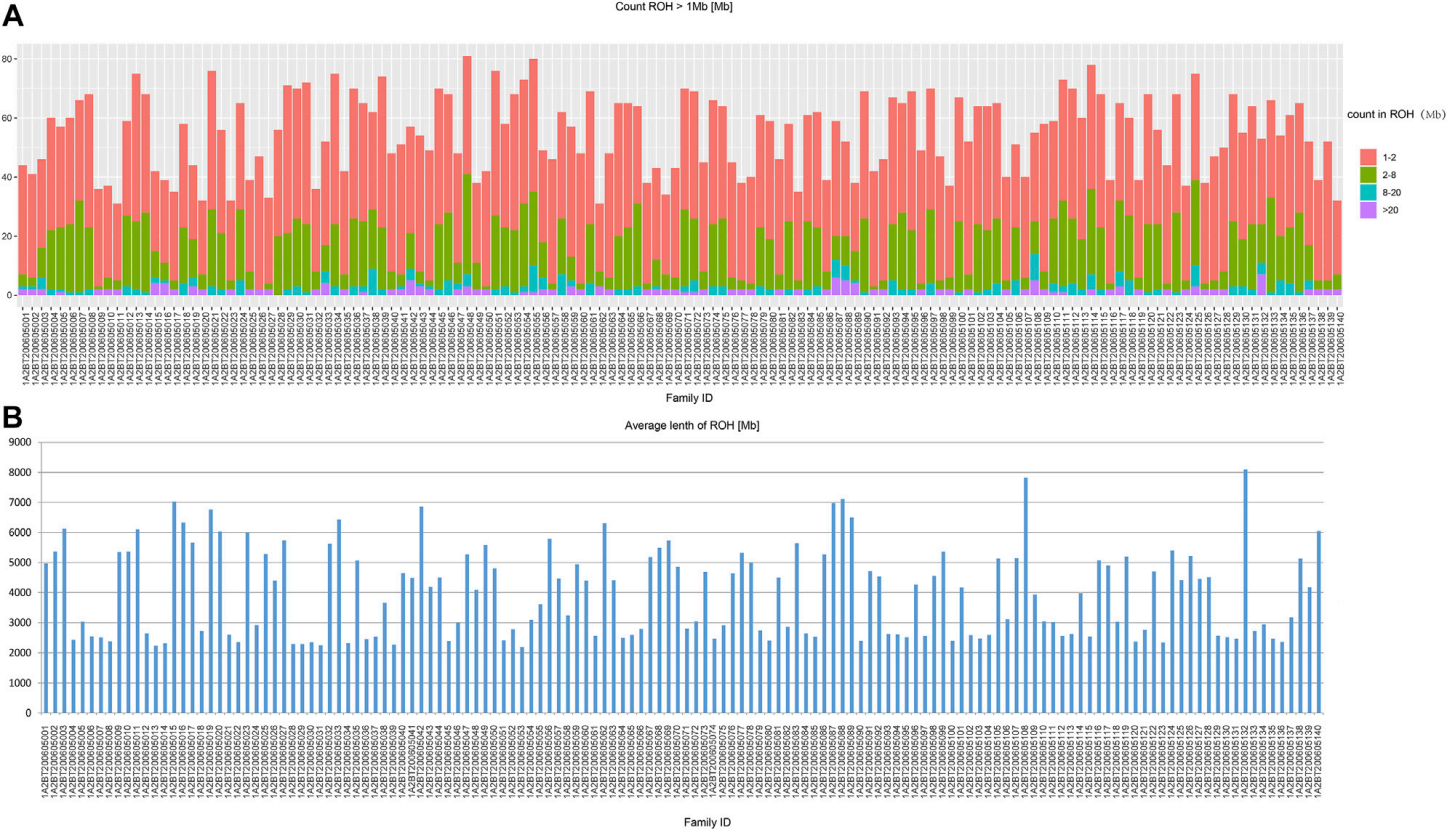
The results of ROH calculation. **(A)** the length distribution; **(B)** the average length.

In the beginning of the population genetic analysis, we presented the results of ROH computation (Figure 2). In our studied populations in Northwest China, the ROH segments were mainly short fractions which were between 1 and 2 Mb. And the long fractions which were longer than 20 Mb were rare. Therefore, our studied populations were not consanguineous communities.

**FIGURE 3:**
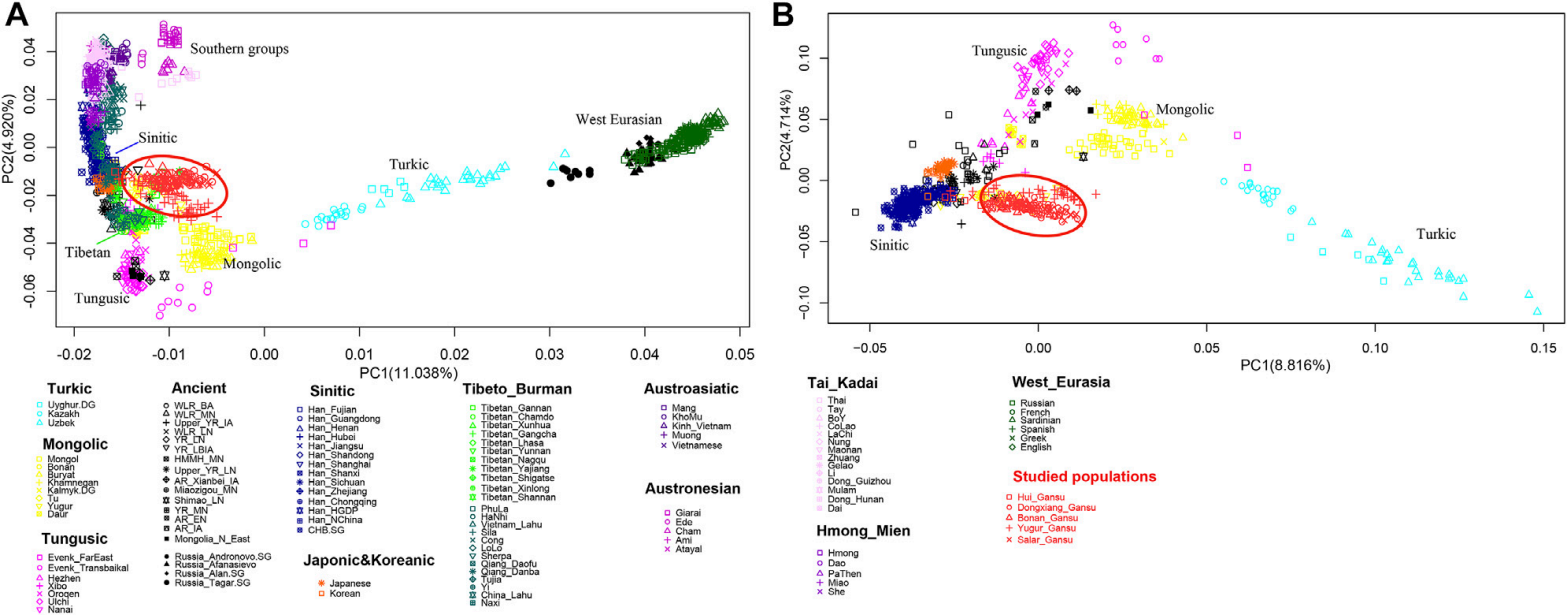
Patterns of genetic relationship among published East Asian populations and our newly genotyped five populations inferred from the principal component analysis. **(A)** East Asians including southern populations and with the West Eurasians; **(B)** East Asians without southern populations and without the West Eurasians.

We firstly conducted PCA to infer the general genetic structure of our sampled populations with other East Asians (Figure 3). From the PCA plot, we found the genetic clusters were consistent with the geographic, and linguistic categories in East Asia. We observed the following clear genetic clusters or clines. A genetic cline related to Turkic speaking populations, which was driven by populations with a large amount of West Eurasian related ancestry, such as Uyghur and Uzbek ethnic groups; a cluster with the Mongolic speaking populations; a cluster related to Tungusic speaking populations; a cluster of populations in West Eurasia. A cluster of Tibetan populations on the high-altitude region; a cluster with Han Chinese groups; and a huge cluster related to southern populations in East Asia speaking Hmong-Mien, Austroasiatic, Tai-Kadai, and Austronesian languages. Our newly reported samples in Gansu province clustered genetically between the Han Chinese groups and the Turkic speaking populations. We next removed the populations from southern China and Southeast Asia and the human groups in West Eurasia to show a more clearly clustering pattern among northern populations. In the zoomed PCA, our newly reported populations were close to the Han Chinese cluster, but also shifted towards the Turkic genetic cline, showing genetic affinity with both Turkic populations, and Han Chinese.

**FIGURE 4:**
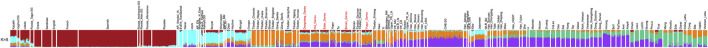
ADMIXTURE analysis result visualization at K = 5 as the corresponding cross-validation error was the lowest. And our studied populations in Gansu were marked by red color.

We next carried out the model-based ADMIXTURE clustering analysis. We observed the lowest CV error at K = 5. We then made the visualization of the result at K = 5 with five colors (Figure 4): The red component was primarily enriched in West Eurasians; the blue component was largely shown in the Mongolic and Tungusic speaking populations; the orange component was mainly detected in the Tibetan groups; the green component was largely presented in Austronesian speaking populations; the purple component was mainly enriched in some southern groups in East Asia. Our newly reported Hui, Dongxiang, Bonan, Yugur, and Salar samples harbored large orange and purple ancestral component related to East Asia and a part of red ancestral component related to the West Eurasia. The ancestry assignment was consistent with previous PCA analysis.

**FIGURE 5:**
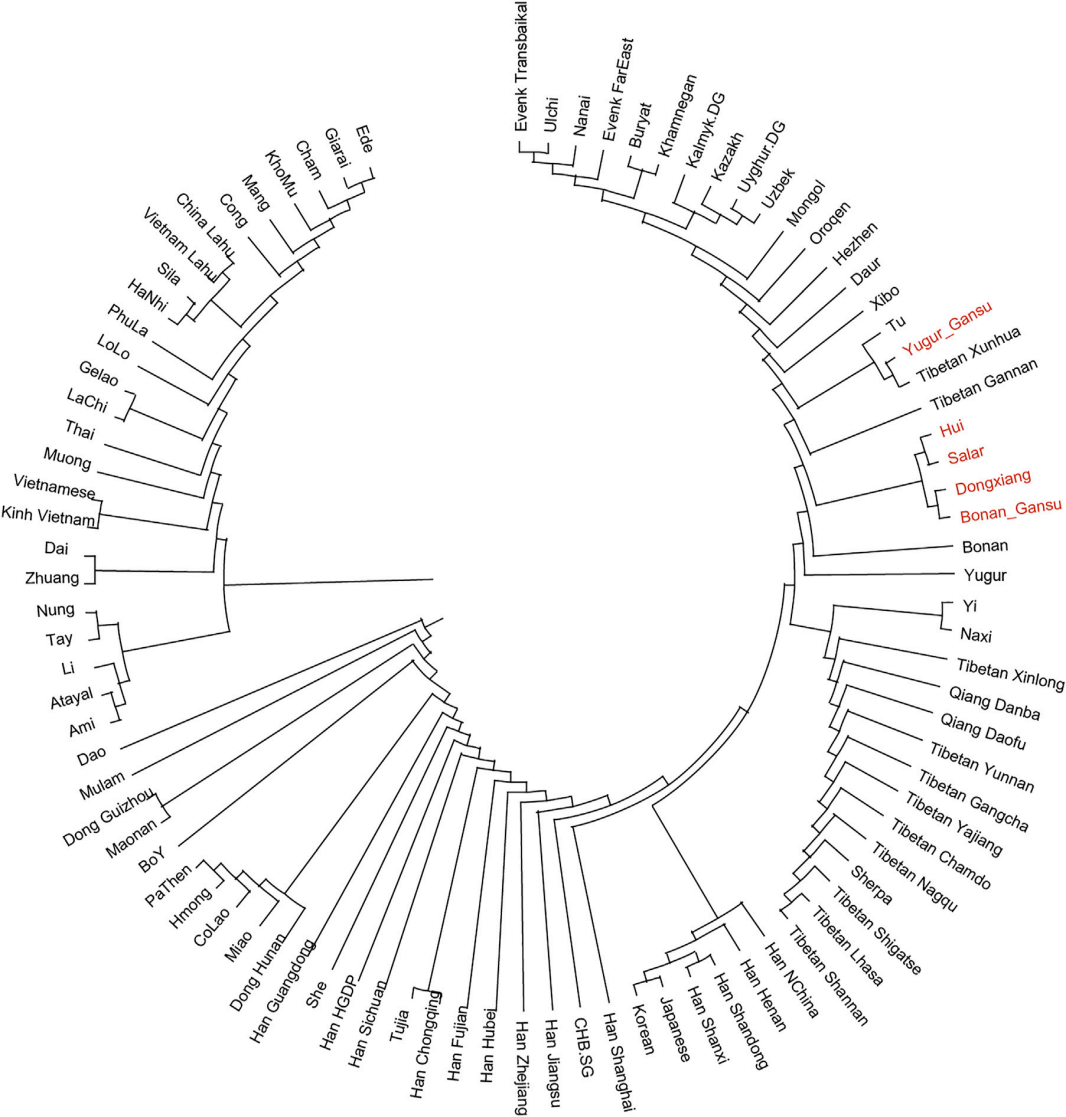
Phylogenetic tree among our studied populations in Gansu and reference populations in Eurasia. Our samples in Gansu province were marked with red color.

In the following, we calculated the pairwise Fst values for our studied populations in Gansu province together with reference populations in Eurasia and constructed a phylogenetic tree (Figure 5). In this phylogenetic tree, our newly reported groups in Gansu province clustered closely with the surrounding Altaic speaking populations in northern China. Notablely, The Yugur group clustered together with Tibetans from Xunhua and Gannan and Tu.

**FIGURE 6:**
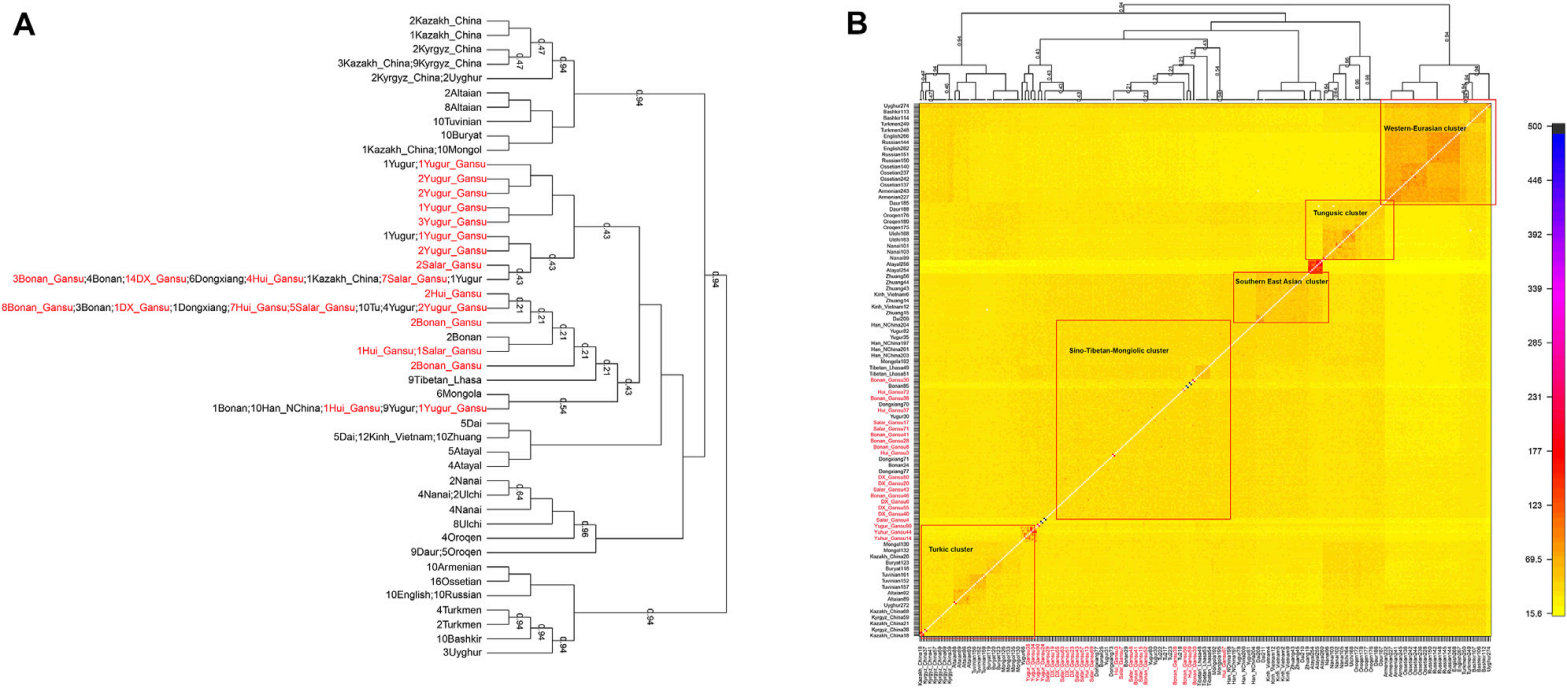
The heat map of sharing haplotypes and clustering dendrogram by fineSTRUCTURE. DX = Dongxiang. **(A)** the dendrogram. **(B)** the heat map of sharing haplotypes.

Next, we characterized the finer-scale population structure of our studied groups in Gansu by the haplotype-based fineSTRUCTURE. The inferred polygenetic tree based on the linked coancesty matrix showed all populations were clustered well according to geographical positions and language classification. Overall, our studied population clustered with published Mongolic speakers and Turkic speakers Kazakh in China, forming the major branch that also included Han, Tibetan, and Mongolia of China. Our Yugur_Gansu population formed relatively sporadic and formed serval small branches, even one individual clustered with published Yugur (Figure 6A). In addition, Hui people clustered with Bonan, Dongxiang, Salar, Yugur groups. Heatmap (Figure 6B) and the corresponding clustering patterns showed five major clusters, the Sino-Tibetan-Mongolic cluster included Chinese Mongolic populations in northwestern China, Tibetan and Han populations, our studied populations the larger amount of haplotype sharing among those populations.

### Continuity and Admixture of Populations by the Allele-Shared *f-* Statistics

**FIGURE 7:**
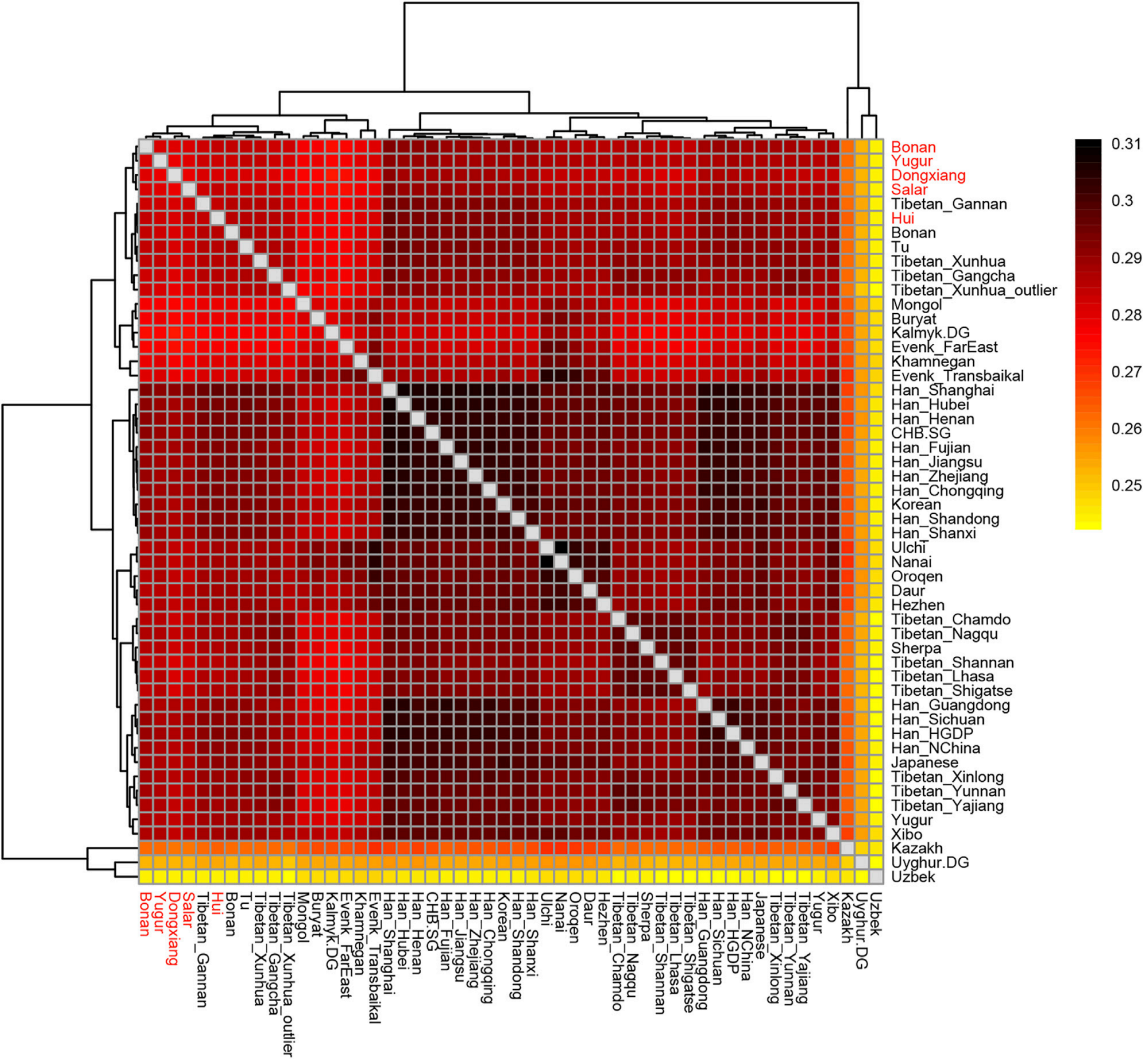
Heatmap results of the outgroup-*f*_*3*_ statistics of the form *f*_*3*_ (X, Y; Mbuti). The larger values indicated that they shared more genetic drifts. Here the Outgroup was Mbuti.

In the following, we calculated the outgroup-*f*_*3*_ statistics in the form of *f*_*3*_ (*X*, *Y*; *Mbuti*) to quantify the population differentiation across East Asia. We showed the results in a heatmap plot (Figure 7). The larger value of the statistics indicated that the two groups shared more genetic drifts after the separation from an African outgroup. We found the majority of Han Chinese populations shared more alleles with each other and clustered together. The Mongolic and Tungusic populations (Ulchi, Nanai, Oroqen, Daur, Hezhen) also clustered together. Our studied populations Hui, Dongxiang, Bonan, Yugur, and Salar clustered together and shared more genetic drifts with Han Chinese populations than with Tibetan groups.

In addition, we performed the admixture-*f*_*3*_ statistics in the form of *f*_*3*_ (Source1, Source2; Target) to explore the possible ancestral source populations for our studied populations in Gansu province. We observed the most significant negative signals when using the Neolithic Yellow River farming groups and the Bronze Age to Iron Age Steppe groups from West Eurasia and Central Asia as sources (Table 1), suggesting the gene flow from West Eurasia into northwest China.

**TABLE 1:** Admixture *f3* statistics of the form (Source1, Source2; Target) with the lowest *f3* values.

Source 1	Source 2	Target	f_3	Std. err	Z	SNPs
Kazakhstan_Andronovo.SG	Upper_YR_LN	Hui	−0.010305	0.001639	−6.288	55271
Kazakhstan_Andronovo.SG	Upper_YR_IA	Hui	−0.009701	0.001942	−4.995	53232
Kazakhstan_Kangju.SG	Shimao_LN	Hui	−0.009247	0.001029	−8.99	161279
Russia_Alan.SG	WLR_LN	Hui	−0.009221	0.001209	−7.63	136872
Russia_Alan.SG	YR_LBIA	Hui	−0.009003	−0.000714	−12.609	165864
Russia_Alan.SG	WLR_LN	Dongxiang	−0.014067	−0.001139	−12.348	138809
CHB.SG	Anatolia_N	Dongxiang	−0.013755	−0.000316	−43.553	172663
CHB.SG	Russia_MLBA_Sintashta	Dongxiang	−0.013573	0.000324	−41.928	171061
Anatolia_N	YR_LBIA	Dongxiang	−0.013525	−0.000628	−21.527	170054
Kazakhstan_Kangju.SG	Shimao_LN	Dongxiang	−0.013429	−0.00097	−13.847	163617
Russia_MLBA_Sintashta	Miaozigou_MN	Bonan	−0.010536	0.001382	−7.621	49714
Kazakhstan_Andronovo.SG	Upper_YR_LN	Bonan	−0.010432	0.001621	−6.436	55987
Russia_Alan.SG	WLR_LN	Bonan	−0.010298	0.001191	−8.645	138290
Kazakhstan_Kangju.SG	Shimao_LN	Bonan	−0.010185	−0.000996	−10.226	163067
CHB.SG	Anatolia_N	Bonan	−0.010179	−0.000311	−32.782	172482
Kazakhstan_Andronovo.SG	Upper_YR_LN	Yugur	−0.008896	0.001651	−5.388	55670
Russia_Alan.SG	Wuzhuangguoliang	Yugur	−0.008814	0.002125	−4.148	28513
Russia_MLBA_Sintashta	Miaozigou_MN	Yugur	−0.008676	0.001367	−6.346	49569
Kazakhstan_Kangju.SG	Shimao_LN	Yugur	−0.00843	0.001004	−8.393	162332
Kazakhstan_Andronovo.SG	Upper_YR_IA	Yugur	−0.008279	0.001914	−4.326	53651
Kazakhstan_Andronovo.SG	Upper_YR_LN	Salar	−0.011922	0.001611	−7.4	55593
Kazakhstan_Andronovo.SG	Upper_YR_IA	Salar	−0.011297	0.001916	−5.897	53574
Kazakhstan_Kangju.SG	Shimao_LN	Salar	−0.011024	−0.001022	−10.781	162165
Russia_Alan.SG	WLR_LN	Salar	−0.010966	0.001182	−9.28	137507
Russia_Alan.SG	Shimao_LN	Salar	−0.010849	−0.000996	−10.893	165726

### The Ancestry Inference of the Populations in Northwest China

**FIGURE 8:**
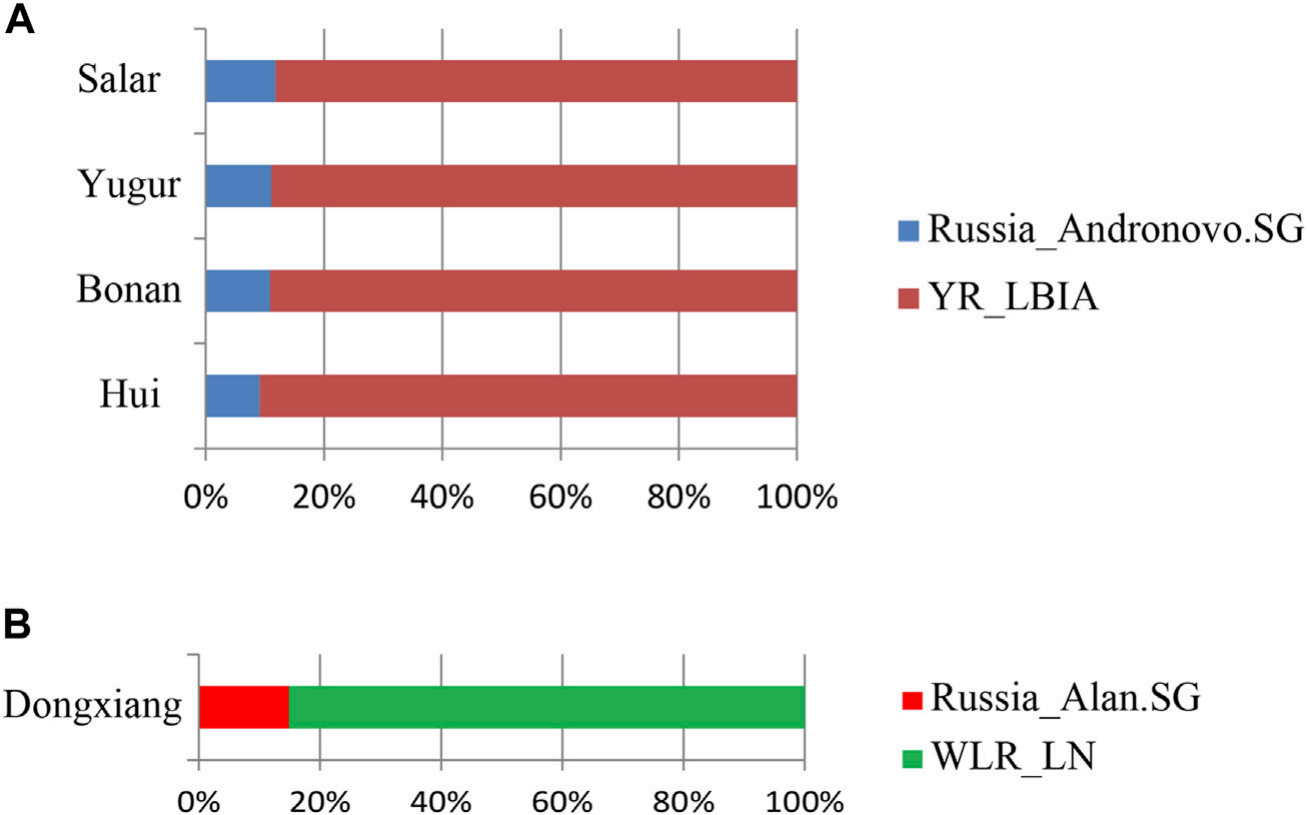
qpAdm based admixture models for the populations in our study in Gansu province. The 2-way admixture models for our Gansu samples were presented when the *p* values >0.05 at the rank = 1. **(A)** Hui, Bonan, Yugur, Salar ethnic groups. **(B)** Dongxiang ethnic group.

We next carried out *qpAdm* analysis to infer the admixture proportions in our studied Gansu populations (Figure 8; Table 2). The eastern ancestral source populations we selected were the Yellow River farming groups from the Bronze Age to Iron Age, and the western ancestral source populations we selected were ancient populations of Andronovo and Alan cultures, since theyprovided the most significant negative admixture-*f*_*3*_ values. We used the following set of populations as outgroups: Mbuti, Russia_EBA_Yamnaya_Samara, Anatolia_N, Russia_MA1, Russia_Afanasievo, Mongolia_N_East, Ust_Ishim, Russia_Kostenki14, Iran_C_SehGabi. Our studied populations could be modeled by two-way admixture with the *p*-value > 0.05 at rank = 1. We estimated the genetic proportions of Russia_Andronovo related ancestry were 9.1 ∼ 11.8%, while the genetic proportions of YR_LBIA farming group related ancestry were 88.2 ∼ 90.9% in Hui, Bonan, Yugur, and Salar groups. Given the pair groups consisting of Late Neolithic farmers in West Liao River (WLR_LN) and Iron Age Alan people in Russia (Russia_Alan) in admixture *f*_*3*_ showed the most significant admixture signal, we found that the Dongxiang group derived 14.9% western Eurasian ancestry from Russia_Alan related groups and the left from WLR_LN related groups. In general, the qpAdm model indicated the west-east admixture in our five studied populations, showing East Asian related ancestry dominantly made contribution to the genetic formation of Northwest Chinses Altaic speaking groups with different proportions of West Eurasian related ancestry.

**TABLE 2:** Two-way *qpAdm* models of studied populations in Gansu.

Studied population	Proportion	Std. err	Proportion	Std. err	*p* value
	Russia_Andronovo.SG		YR_LBIA		
Hui	0.091	0.007	0.909	0.007	0.206
Bonan	0.109	0.007	0.891	0.007	0.0536
Yugur	0.111	0.007	0.889	0.007	0.546
Salar	0.118	0.007	0.882	0.007	0.098
	Russia_Alan.SG		WLR_LN		
Dongxiang	0.149	0.011	0.851	0.011	0.304

### Y Chromosomal and MtDNA Haplogroup Assignment

We assigned the haplogroups of Y chromosome and mtDNA for our newly genotyped samples (Table 3). The haplogroup R1a1a1b2 was the most frequent patrilineal lineage in the Hui, Bonan, and Salar groups. We also detected haplogroup D1a1a1a1a2a∼, H1a1a1a, J2a1a, J2a1h2b, J2a2, N1a2b3, O2a2a1a2a1a, and O2a2b1a1a6b in our Hui samples. Haplogroup D1a1a1a1a2a∼ and O2a2b1a1a were also found in Bonan group. The haplogroup O1b1a1a1b2 was also presented in Salar group. Haplogroup J2a1h2, which was mostly found in the Middle East, was the most prevailing lineage in Dongxiang people. We also found D1a1a1a2, O2a2b1a1a6, and R2a2 in the Dongxiang group. Haplogroups C2b1a1, D1a1a1a1a2a∼, O2a2b1a2a1a2, O2a2b1a2b2, and Q1b2b1b2b2∼ were the prevalent lineages in the studied Yugur group. The distribution of Y haplotype indicated the influence of westward expansion of several ancestral sources in genetic formation of Northwest Chinese Altaic populations, including West Eurasian, Sino-Tibetan, common ancestor of Altaic related ancestry.

**TABLE 3:** The Y-chromosome haplogroups distribution of our studied populations.

	Y Haplogroup	Frequency
Hui	D1a1a1a1a2a∼	0.100
	H1a1a1a	0.100
	J2a1a	0.100
	J2a1h2b	0.100
	J2a2	0.100
	N1a2b3	0.100
	O2a2a1a2a1a	0.100
	O2a2b1a1a6b	0.100
	R1a1a1b2	0.200
Dongxiang	D1a1a1a2	0.133
	E1b1a1a1a2a1a3b1a10b∼	0.067
	J2a1h2	0.200
	J2a2	0.067
	L1a2a1b2∼	0.067
	N1a1a1a1a3a2a∼	0.067
	N1a3∼	0.067
	O2a2b1a1a6	0.133
	R1a1a1b2	0.067
	R2a2	0.133
Bonan	C2b1a2a2a∼	0.0625
	D1a1a1a1a2a∼	0.125
	D1a2a1∼	0.0625
	J2a1h2	0.0625
	N1a2b3a∼	0.0625
	O1b1a1a1a1b1b	0.0625
	O1b1a1a1a2	0.0625
	O2a2b1a1a	0.125
	O2a2b1a2a1d	0.0625
	Q1b1a3b1a1∼	0.0625
	Q2a1c1b1∼	0.0625
	R1a1a1b2	0.1875
Yugur	C2b1a1	0.133
	C2b1a3b∼	0.067
	D1a1a1a1a2a∼	0.133
	D1a1a1a2	0.067
	O2a2b1a1a6	0.067
	O2a2b1a2a1a2	0.133
	O2a2b1a2b2	0.133
	O2a2b2a2a1	0.067
	Q1b1a3a∼	0.067
	Q1b2b1b2b2∼	0.133
Salar	I2a2a1b2a1b1b2a2∼	0.091
	J2a1	0.091
	N1b2a2∼	0.091
	O1b1a1a1b2	0.182
	O2a1a1b1a2	0.091
	O2a1c1a1a1a1a1b1a∼	0.091
	O2a2b1a2a1a1a1	0.091
	R1a1a1b2	0.273

We next assigned the matrilineal mtDNA haplogroups for our studied populations. In the Hui group, we observed diverse mtDNA haplogroups, including D4, D5a2a1, F1, G3a1′2, M7, M8, Z3, and Z4. The maternal profile of Dongxiang group was similar to that in the Hui group, but the haplogroup A, B4, and F2 were more prevalent in Dongxiang. We found D4 was the most dominant lineage in Bonan group and we also detected B and G2a in Bonan group. Haplogroup D4 was also the most dominant haplogroup in Yugur group, following by A1, C4, F1g, and M9a1 haplogroups. Haplogroup A was the most prevailing haplogroup in the Salar group, following by F1, M9a1b1, and Z3. The main mtDNA haplogroups in our samples were also prevalent in East Asia, suggesting the local East Asians largely contributed to the maternal gene pool of Gansu Altaic speaking populations. The genetic influence from the West Eurasian human populations were more significant in the patrilineal lineages than in the matrilineal lineages. The details of the distribution of mtDNA haplogroups were listed in Table 4.

**TABLE 4:** The mtDNA haplogroups distribution for our studied populations.

Hui		Dongxiang		Bonan		Yugur		Salar	
Haplogroup	Frequency	Haplogroup	Frequency	Haplogroup	Frequency	Haplogroup	Frequency	Haplogroup	Frequency
A16	0.041666667	A	0.06666667	A	0.03333333	A1	0.1	A	0.115385
B4c1b2c	0.041666667	A1	0.1	B4	0.06666667	A6b	0.033333333	A18	0.038462
B5b2	0.041666667	A6b	0.03333333	B5	0.06666667	B4a3	0.033333333	A5b1b	0.038462
C4d	0.041666667	B4	0.1	B6a	0.03333333	C4	0.1	A8a	0.038462
D4	0.083333333	C5d2	0.03333333	C4	0.1	D4	0.4	B4b1a2a	0.038462
D5	0.041666667	D4	0.1	C5b1b	0.03333333	D5a2a1	0.033333333	C4d	0.038462
D5a2a1a1	0.04166667	D5	0.06666667	D4	0.2	F1g	0.1	D4	0.076923
F1	0.125	F1	0.06666667	F1g	0.03333333	M9a1a1c1b1a	0.066666667	F1	0.115385
F4a2	0.041666667	F2	0.1	G2	0.1	M9a1b1	0.033333333	F3a1	0.038462
G3a1′2	0.083333333	F4b	0.03333333	H	0.06666667	R9b1a3	0.033333333	G1a1	0.038462
M7	0.125	H15	0.03333333	M10a1a1b	0.03333333	U4b1a1a1	0.033333333	G2a	0.076923
M8	0.083333333	H5	0.06666667	M7b1a	0.03333333	U7a	0.033333333	H7b1	0.038462
N9a2	0.04166667	M7b1a1a3	0.03333333	M8	0.06666667			M11a2	0.038462
Z3	0.083333333	M8	0.06666667	M9a1a1c1a	0.03333333			M21b	0.038462
Z4	0.083333333	T2a1a	0.03333333	X2b4	0.03333333			M9a1b1	0.115385
		X2	0.03333333	Z3a	0.06666667			Z3	0.115385
		Y1b1a	0.03333333						

### The Admixture Time Estimation for the Populations in Northwest China

We estimated the admixture time between the East and West Euraisan related ancestry in Northwest Chinese populaton using the weighted linkage disequilibrium-based admixture inference implemented in ALDER (Loh et al., 2013). We used Han_HGDP and Sardinian as two ancestral surrogates to calculated the east-west admixture time and listed the results in Table 5. The average admixture time calculated by the 2-ref weighted LD for our five studied populations ranged from 25 to 31 generations, which was approximately 750–930 years before present assuming 30 years one generation (Table 5). The east-west interactions were suggested to have occurred in about the Song and Yuan Dynasty of China.

**TABLE 5:** The admixture time estimation by ALDER for our studied populations.

Population	1-Ref weighted LD with weights Sardinian (generation)	Z-score	1-Ref weighted LD with weights Han_HGDP (generation)	Z-score	2-Ref weighted LD with weights Sardinian and Han_HGDP (generation)	Z-score
Hui	34.98 ± 4.20	8.32	97.35 ± 35.92	2.71	31.36 ± 3.27	9.58
Dongxiang	28.71 ± 2.60	11.03	40.77 ± 7.40	5.51	26.73 ± 2.61	10.24
Bonan	33.21 ± 2.42	13.72	-	-	26.08 ± 2.50	10.42
Yugur	33.65 ± 4.53	7.42	-	-	25.32 ± 3.81	6.65
Salar	25.70 ± 3.64	7.07	33.74 ± 11.91	2.83	24.77 ± 3.83	6.47

We further performed haplotype-based GLOBETROTTER to obtain the admixture landscaped of our studied northwestern Chinese populations (Table 6). The east-west admixture could be traced back to ∼21 to ∼25 generations ago (approximately ∼630–750 years ago assuming 30 years one generations), with inferring western Eurasian related ancestry represented by English ranging from 16 to 24%, coinciding with the results from ALDER. In addition, we observed the minor southern population admixture in Hui, Yugur and Salar (0.2, 0.06, and 0.04, respectively).

**TABLE 6:** The admixture events of our studied populations by GLOBETROTTER.

Recipient.Population	Model	Gen.1date	Proportion.source1	Bestmatch.event1.source1	Bestmatch.event1.source2	Proportion.event2.source1	Bestmatch.event2.source1	Bestmatch.event2.source2	MaxR2fit.1date	Fit.quality.1event	Fit.quality.2events	Gen.2dates.date1	Gen.2dates.date2	Proportion.date1.source1	Bestmatch.date1.source1	Bestmatch.date1.source2	Proportion.date2.source1	Bestmatch.date2.source1	Bestmatch.date2.source2	MaxScore.2events
Hui_Gansu	1-DATE	24.88801703	0.18	English	Han_NChina	0.2	Kinh_Vietnam	Salar_Gansu	0.920102825	0.99996966	0.999996517	1.000004327	24.44514919	0.42	Atayal	Salar_Gansu	0.18	English	Han_NChina	0.111809238
Dongxiang_Gansu	1-DATE	21.43093694	0.24	English	Han_NChina	0.41	Mongol	Dongxiang	0.943671053	0.999996881	0.999999612	1.000023459	26.76153143	0.13	Turkmen	Tu	0.24	English	Han_NChina	0.154655956
Bonan_Gansu	multiple-dates	24.95247758	0.19	English	Han_NChina	0.49	Yugur	Bonan	0.918212959	0.999969001	0.9999979	8.270277733	30.57366368	0.34	Uyghur.DG	Bonan	0.18	English	Han_NChina	0.478201465
Yugur_Gansu	1-DATE	23.47510082	0.16	English	Yugur	0.06	Atayal	Tibetan_Lhasa	0.884253347	0.999991289	0.999998974	11.71119508	44.60320405	0.07	English	Tu	0.13	English	Yugur	0.073485128
Salar_Gansu	1-DATE	20.80295557	0.2	English	Han_NChina	0.04	Atayal	Hui_Gansu	0.943365942	0.99999999	1	10.74070225	43.5990788	0.07	English	Hui_Gansu	0.16	English	Han_NChina	0.176636782

## Discussion

The East Asia is a region with diverse culture communications, multiple language interactions, and complex population history. Many previous studies provided that the genetic substructure of populations in East Asia was consistent with the language affinities. The Hexi Corridor and its surrounding regions were known for the famous Majiayao civilization in the middle and late Neolithic Age and subsequently controlled by the Rong-Di tribes before the Han Dynasty. Moreover, the Northwest China witnessed the intersection of the eastward expansion of the barley and wheat agriculture and the westward expansion of the millet agriculture in the Neolithic to Bronze Age. Gansu province isone of the key regions in Northwest China where also connects the Hexi Corridor and Tibetan-Yi Corridor. The genetic diversity, fine-scale genetic substructure, and the western Eurasian admixture in the populations of Gansu are still needed to be fully explored. We collected 140 modern individuals from Hui, Dongxiang, Bonan, Yugur, and Salar groups from the Gansu province and genotyped with genome-wide SNPs. We reconstructed the population admixture history of the Altaic speaking populations in northwest China.

Our studied populations of Northeast China showed similar genetic profile among those populations, suggesting the relatively genetic homogeneity in Northwest China, even though harboring subtle different proportions of East, and West Eurasian related ancestry. The close genetic affinity among Chinese Turkic speakers, Tungusic, and Mongolic populations indicated the probability of common ancestor of Altaic speakers. Our results showed that both West and East Eurasian contributed the genetic formation of Altaic populations in Northwest China, which coinciding with previous studies suggested the east-west admixture in Alatic populations and Hui population (Xu and Jin, 2008; Bai et al., 2018; Jeong et al., 2019; Zhao et al., 2020; Ma et al., 2021). The closer genetic relationship between our studied population and Sino-Tibetan populations and the results of qpAdm and GLOBETROTTER suggested the majority contributing East Eurasian ancestry might derived from millet farmers in Yellow River Basin related population. The eastward expansion of Bronze Age West Steppe nomadic groups limitedly impacted the gene pool of the East Eurasian. The five studied Altaic speaking groups were suggested to harbored the lower proportion of Middle and Late Bronze West Steppe pastoralists represented by Andronovo culture. This was also supported by the high frequencies of Y chromosomal haplogroup R1a1a1b2 which prevailed Middle and Late Bronze Age Steppe populations in Hui, Bonan, and Salar groups (Narasimhan et al., 2019). The genetic admixture from West Eurasians was probably driven by male dominant migration which showing the higher frequencies of West Eurasian related paternal Y chromosome lineages and the absence of maternal mtDNA lineage related to West Eurasian. The paleogenomic studies exhibited the most complex pattern of male-biased admixture in the demographic dynamics of the East Steppe (Jeong et al., 2020).

Considering that the West Eurasian related ancestry proportions were limited in our studied populations (<15%), we noted that it was hard to determine the exact genetic source for the admixture. The sequencing of more ancient genomes from Northwest China may shed more light on determining the West Eurasian sources. We estimated the admixture event to have occurred in historic period based on ALDER and GLOBETROTTER (approximately dating to ∼750–930 years ago, ∼630–750 years ago, respectively). The ancient admixture we identified was roughly corresponding to the Song to Yuan Dynasty. But we noted if the admixture did not happen immediately after arrival or multiple times over an extended period, however, the true start of admixture would have been more ancient. Furthermore, the intensive and continuous contact between West and East Eurasian population started as early as the Bronze Age due to the advantage of horses, and the interaction became more frequent with the opening of Silk Road in the Han Dynasty. The establishment of Mongols empire and the Mongolian Conquests in the 13th and 14th centuries facilitated the west-east contacts. The true admixture history in Northwest China could be more complex than the simplified models as we presented in this study, the populations studied here, however, harbored prominent local East Eurasian related ancestry and limited West Eurasian related ancestry.

Running through the ancient Silk Road, the human groups were all presented a west-east admixture structure. The Uyghur in Xinjiang was a typical one. Besides, the Altaic speaking populations in Central Asia all have the west-east interactions in genetic structure and culture. The east endpoint of the ancient Silk Road was near Chang’an City, and the Gansu pathway was the only route to it. The Altaic populations in this region lack of large-scale sampling and genome-wide genetic analysis. Our research answered this issue at a certain degree, but the more elaborate admixture history needed to be explored from the whole genome sequencing next.

## Data Availability

The datasets presented in this study can be found in online repositories. The names of the repository/repositories and accession number(s) can be found below: https://zenodo.org/, https://doi.org/10.5281/zenodo.5542715.

## References

[B1] Abelson A. (1978). Population Structure in the Western Pyrenees: Social Class, Migration and the Frequency of Consanguineous Marriage, 1850 to 1910. Ann. Hum. Biol. 5, 165–178. 10.1080/03014467800002761 655627

[B2] Alexander D. H. Novembre J. Lange K. (2009). Fast Model-Based Estimation of Ancestry in Unrelated Individuals. Genome Res. 19, 1655–1664. 10.1101/gr.094052.109 19648217 PMC2752134

[B3] Antwerpen M. Beyer W. Bassy O. Ortega-Garcia M. V. Cabria-Ramos J. C. Grass G. (2019). Phylogenetic Placement of Isolates within the Trans-eurasian Clade A.Br.008/009 of Bacillus Anthracis. Microorganisms 7, 689. 10.3390/microorganisms7120689 PMC6955976 31842497

[B4] Bai F. Zhang X. Ji X. Cao P. Feng X. Yang R. (2020). Paleolithic Genetic Link between Southern China and Mainland Southeast Asia Revealed by Ancient Mitochondrial Genomes. J. Hum. Genet. 65, 1125–1128. 10.1038/s10038-020-0796-9 32653893

[B5] Bai H. Guo X. Narisu N. Lan T. Wu Q. Xing Y. (2018). Whole-genome Sequencing of 175 Mongolians Uncovers Population-specific Genetic Architecture and Gene Flow throughout North and East Asia. Nat. Genet. 50, 1696–1704. 10.1038/s41588-018-0250-5 30397334

[B6] Bento C. B. Filoso S. Pitombo L. M. Cantarella H. Rossetto R. Martinelli L. A. (2018). Impacts of Sugarcane Agriculture Expansion over Low-Intensity Cattle Ranch Pasture in Brazil on Greenhouse Gases. J. Environ. Manage. 206, 980–988. 10.1016/j.jenvman.2017.11.085 29223108

[B7] Cao Y. Li L. Li L. Xu M. Feng Z. Sun X. (2020). The ChinaMAP Analytics of Deep Whole Genome Sequences in 10,588 Individuals. Cell Res 30, 717–731. 10.1038/s41422-020-0322-9 32355288 PMC7609296

[B8] Chang C. C. Chow C. C. Tellier L. C. Vattikuti S. Purcell S. M. Lee J. J. (2015). Second-generation PLINK: Rising to the challenge of Larger and Richer Datasets. GigaSci 4, 7. 10.1186/s13742-015-0047-8 PMC4342193 25722852

[B9] Chen P. Zou X. Wang M. Gao B. Su Y. He G. (2019). Forensic Features and Genetic Structure of the Hotan Uyghur Inferred from 27 Forensic Markers. Ann. Hum. Biol. 46, 589–600. 10.1080/03014460.2019.1687751 31762339

[B10] Cheng T. O. (1985). Medicine and Health Care along the Silk Road. Arch. Intern. Med. 145, 137–138. 10.1001/archinte.1985.00360010175029 3970625

[B11] Coulehan J. (2020). Cultural Exchange. Ann. Intern. Med. 172, 158. 10.7326/m19-0932 31958835

[B12] Damgaard P. d. B. Marchi N. Rasmussen S. Peyrot M. Renaud G. Korneliussen T. (2018). 137 Ancient Human Genomes from across the Eurasian Steppes. Nature 557, 369–374. 10.1038/s41586-018-0094-2 29743675

[B13] De Barros Damgaard P. Martiniano R. Kamm J. Moreno-Mayar J. V. Kroonen G. Peyrot M. (2018b). The First Horse Herders and the Impact of Early Bronze Age Steppe Expansions into Asia. Science 360, eaar7711. 10.1126/science.aar7711 29743352 PMC6748862

[B14] De Barros Damgaard P. Marchi N. Rasmussen S. Peyrot M. Renaud G. Korneliussen T. (2018a). Author Correction: 137 Ancient Human Genomes from across the Eurasian Steppes. Nature 563, E16. 10.1038/s41586-018-0488-1 30166629

[B15] Delaneau O. Zagury J.-F. Marchini J. (2013). Improved Whole-Chromosome Phasing for Disease and Population Genetic Studies. Nat. Methods 10, 5–6. 10.1038/nmeth.2307 23269371

[B16] Duan S.-F. Han P.-J. Wang Q.-M. Liu W.-Q. Shi J.-Y. Li K. (2018). The Origin and Adaptive Evolution of Domesticated Populations of Yeast from Far East Asia. Nat. Commun. 9, 2690. 10.1038/s41467-018-05106-7 30002370 PMC6043522

[B17] Elfari M. Schnur L. F. Strelkova M. V. Eisenberger C. L. Jacobson R. L. Greenblatt C. L. (2005). Genetic and Biological Diversity Among Populations of Leishmania Major from Central Asia, the Middle East and Africa. Microbes Infect. 7, 93–103. 10.1016/j.micinf.2004.09.010 15716069

[B18] Feng Q. Lu Y. Ni X. Yuan K. Yang Y. Yang X. (2017). Genetic History of Xinjiang's Uyghurs Suggests Bronze Age Multiple-Way Contacts in Eurasia. Mol. Biol. Evol. 34, 2572–2582. 10.1093/molbev/msx177 28595347

[B19] Feng R. Zhao Y. Chen S. Li Q. Fu Y. Zhao L. (2020). Genetic Analysis of 50 Y-STR Loci in Dong, Miao, Tujia, and Yao Populations from Hunan. Int. J. Leg. Med 134, 981–983. 10.1007/s00414-019-02115-z 31263947

[B20] Fu Q. Meyer M. Gao X. Stenzel U. Burbano H. A. Kelso J. (2013). DNA Analysis of an Early Modern Human from Tianyuan Cave, China. Proc. Natl. Acad. Sci. 110, 2223–2227. 10.1073/pnas.1221359110 23341637 PMC3568306

[B21] Goldberg A. Günther T. Rosenberg N. A. Jakobsson M. (2017). Reply to Lazaridis and Reich: Robust Model-Based Inference of Male-Biased Admixture during Bronze Age Migration from the Pontic-Caspian Steppe. Proc. Natl. Acad. Sci. USA 114, E3875–E3877. 10.1073/pnas.1704442114 28476765 PMC5441823

[B22] Haak W. Lazaridis I. Patterson N. Rohland N. Mallick S. Llamas B. (2015). Massive Migration from the Steppe Was a Source for Indo-European Languages in Europe. Nature 522, 207–211. 10.1038/nature14317 25731166 PMC5048219

[B23] Heizhati M. Wang L. Yao X. Li M. Hong J. Luo Q. (2020). Prevalence, Awareness, Treatment and Control of Hypertension in Various Ethnic Groups (Hui, Kazakh, Kyrgyz, Mongolian, Tajik) in Xinjiang, Northwest China. Blood Press. 29, 276–284. 10.1080/08037051.2020.1745055 32349556

[B24] Hellenthal G. Busby G. B. J. Band G. Wilson J. F. Capelli C. Falush D. (2014). A Genetic Atlas of Human Admixture History. Science 343, 747–751. 10.1126/science.1243518 24531965 PMC4209567

[B25] Hemphill B. E. Mallory J. P. (2004). Horse-mounted Invaders from the Russo-Kazakh Steppe or Agricultural Colonists from Western Central Asia? A Craniometric Investigation of the Bronze Age Settlement of Xinjiang. Am. J. Phys. Anthropol. 124, 199–222. 10.1002/ajpa.10354 15197817

[B26] HUGO Pan-Asian SNP Consortium Abdulla M. A. Ahmed I. Assawamakin A. Bhak J. Brahmachari S. K. (2009). Mapping Human Genetic Diversity in Asia. Science 326, 1541–1545. 10.1126/science.1177074 20007900

[B27] Hyten D. L. Cannon S. B. Song Q. Weeks N. Fickus E. W. Shoemaker R. C. (2010). High-throughput SNP Discovery through Deep Resequencing of a Reduced Representation Library to Anchor and orient Scaffolds in the Soybean Whole Genome Sequence. BMC Genomics 11, 38. 10.1186/1471-2164-11-38 20078886 PMC2817691

[B28] Jeong C. Balanovsky O. Lukianova E. Kahbatkyzy N. Flegontov P. Zaporozhchenko V. (2019). The Genetic History of Admixture across Inner Eurasia. Nat. Ecol. Evol. 3, 966–976. 10.1038/s41559-019-0878-2 31036896 PMC6542712

[B29] Jeong C. Wang K. Wilkin S. Taylor W. T. T. Miller B. K. Bemmann J. H. (2020). A Dynamic 6,000-Year Genetic History of Eurasia's Eastern Steppe. Cell 183, 890–904. 10.1016/j.cell.2020.10.015 33157037 PMC7664836

[B30] Jeong C. Wilkin S. Amgalantugs T. Bouwman A. S. Taylor W. T. T. Hagan R. W. (2018). Bronze Age Population Dynamics and the Rise of Dairy Pastoralism on the Eastern Eurasian Steppe. Proc. Natl. Acad. Sci. USA 115, E11248–e11255. 10.1073/pnas.1813608115 30397125 PMC6275519

[B31] Juras A. Makarowicz P. Chyleński M. Ehler E. Malmström H. Krzewińska M. (2020). Mitochondrial Genomes from Bronze Age Poland Reveal Genetic Continuity from the Late Neolithic and Additional Genetic Affinities with the Steppe Populations. Am. J. Phys. Anthropol. 172, 176–188. 10.1002/ajpa.24057 32297323

[B32] Khan M. N. Kalsoom S. Khan A. A. (2017). Food Exchange List and Dietary Management of Non-communicable Diseases in Cultural Perspective. Pak J. Med. Sci. 33, 1273–1278. 10.12669/pjms.335.13330 29142578 PMC5673747

[B75] Kumar S. Stecher G. Tamura K. (2016). MEGA7: Molecular Evolutionary Genetics Analysis Version 7.0 for Bigger Datasets. Mol. Biol. Evol. 33, 1870–1874. 10.1093/molbev/msw054 27004904 PMC8210823

[B33] Lan T. Lin H. Zhu W. Tellier L. C. A. M. Yang M. Liu X. (2019). Correction to: Deep Whole-Genome Sequencing of 90 Han Chinese Genomes. Gigascience 8, giz001. 10.1093/gigascience/giz001 30801124 PMC6388080

[B34] Lawson D. J. Hellenthal G. Myers S. Falush D. (2012). Inference of Population Structure Using Dense Haplotype Data. Plos Genet. 8, e1002453. 10.1371/journal.pgen.1002453 22291602 PMC3266881

[B35] Lazaridis I. Reich D. (2017). Failure to Replicate a Genetic Signal for Sex Bias in the Steppe Migration into central Europe. Proc. Natl. Acad. Sci. USA 114, E3873–E3874. 10.1073/pnas.1704308114 28476764 PMC5441797

[B36] Liu J. Wu D. Wang T. Ji M. Wang X. (2021). Interannual Variability of Dust Height and the Dynamics of its Formation over East Asia. Sci. Total Environ. 751, 142288. 10.1016/j.scitotenv.2020.142288 33181993

[B37] Liu S. Huang S. Chen F. Zhao L. Yuan Y. Francis S. S. (2018). Genomic Analyses from Non-invasive Prenatal Testing Reveal Genetic Associations, Patterns of Viral Infections, and Chinese Population History. Cell 175, 347–359. 10.1016/j.cell.2018.08.016 30290141

[B76] Loh P. R. Lipson M. Patterson N. Moorjani P. Pickrell J. K. Reich D. (2013). Inferring Admixture Histories of Human Populations Using Linkage Disequilibrium. Genetics 193, 1233–1254. 10.1534/genetics.112.147330 23410830 PMC3606100

[B38] Luo L. Gao H. Yao L. Long F. Zhang H. Zhang L. (2020). Genetic Diversity, Forensic Feature, and Phylogenetic Analysis of Guizhou Tujia Population via 19 X-STRs. Mol. Genet. Genomic Med. 8, e1473. 10.1002/mgg3.1473 32881358 PMC7667307

[B39] Ma J. Sun Q. Zhang X. Du H. (2014). Correlation between the Single Nucleotide Polymorphisms of the Human Phosphodiesterase 4D Gene and the Risk of Cerebral Infarction in the Uygur and Han Ethnic Groups of Xinjiang, China. Exp. Ther. Med. 7, 155–160. 10.3892/etm.2013.1370 24348782 PMC3861451

[B40] Ma X. Yang W. Gao Y. Pan Y. Lu Y. Chen H. (2021). Genetic Origins and Sex-Biased Admixture of the Huis. Mol. Biol. Evol. 38, 3804–3819. 10.1093/molbev/msab158 34021754 PMC8382924

[B41] Malyarchuk B. A. Derenko M. Denisova G. Woźniak M. Rogalla U. Dambueva I. (2016). Y Chromosome Haplotype Diversity in Mongolic-Speaking Populations and Gene Conversion at the Duplicated STR DYS385a,b in Haplogroup C3-M407. J. Hum. Genet. 61, 491–496. 10.1038/jhg.2016.14 26911356

[B42] Maramovich A. S. Kosilko S. A. Innokent'eva T. I. Voronova G. A. Bazanova L. P. Nikitin A. I. (2008). Plague in China. Threat of Transmission to Regions of Siberia and Far East. Zh Mikrobiol Epidemiol. Immunobiol, 95–99. 18368762

[B43] Matsumoto G. I. Friedmann E. I. Gilichinsky D. A. (1995). Geochemical Characteristics of Organic Compounds in a Permafrost Sediment Core Sample from Northeast Siberia, Russia. Proc. NIPR Symp. Antarct Meteorites 8, 258–267. 11542910

[B44] Medjugorac I. Kustermann W. Lazar P. Russ I. Pirchner F. (1994). Marker-derived Phylogeny of European Cattle Supports Demic Expansion of Agriculture. Anim. Genet. 25 (Suppl. 1), 19–27. 10.1111/j.1365-2052.1994.tb00399.x 7943980

[B45] Miller J. G. Akiyama H. Kapadia S. (2017). Cultural Variation in Communal versus Exchange Norms: Implications for Social Support. J. Personal. Soc. Psychol. 113, 81–94. 10.1037/pspi0000091 28240938

[B77] Narasimhan V. M. Patterson N. Moorjani P. Rohland N. Bernardos R. Mallick S. (2019). The Formation of Human Populations in South and Central Asia. Science 365, eaat7487. 10.1126/science.aat7487 31488661 PMC6822619

[B46] Ning C. Li T. Wang K. Zhang F. Li T. Wu X. (2020). Ancient Genomes from Northern China Suggest Links between Subsistence Changes and Human Migration. Nat. Commun. 11, 2700. 10.1038/s41467-020-16557-2 32483115 PMC7264253

[B47] Ning C. Wang C.-C. Gao S. Yang Y. Zhang X. Wu X. (2019). Ancient Genomes Reveal Yamnaya-Related Ancestry and a Potential Source of Indo-European Speakers in Iron Age Tianshan. Curr. Biol. 29, 2526–2532. 10.1016/j.cub.2019.06.044 31353181

[B48] Patterson N. Price A. L. Reich D. (2006). Population Structure and Eigenanalysis. Plos Genet. 2, e190. 10.1371/journal.pgen.0020190 17194218 PMC1713260

[B78] Patterson N. Moorjani P. Luo Y. Mallick S. Rohland N. Zhan Y. (2012). Ancient Admixture in Human History. Genetics 192, 1065–1093. 10.1534/genetics.112.145037 22960212 PMC3522152

[B49] Peel K. A. Talley C. B. (1996). Scientific and Cultural Exchange Trip to China. S C Nurse (1994) 3, 28–29. 10.7748/en.3.4.29.s17 9391480

[B79] Purcell S. Neale B. Todd-Brown K. Thomas L. Ferreira M. A. Bender D. (2007). PLINK: A Tool Set for Whole-Genome Association and Population-Based Linkage Analyses. Am. J. Hum. Genet. 81, 559–575. 10.1086/519795 17701901 PMC1950838

[B50] Robino A. Mezzavilla M. Pirastu N. Dognini M. Tepper B. J. Gasparini P. (2014). A Population-Based Approach to Study the Impact of PROP Perception on Food Liking in Populations along the Silk Road. PLoS One 9, e91716. 10.1371/journal.pone.0091716 24626196 PMC3953580

[B51] Rodin R. E. Dou Y. Kwon M. Sherman M. A. D'gama A. M. Doan R. N. (2021). Author Correction: The Landscape of Somatic Mutation in Cerebral Cortex of Autistic and Neurotypical Individuals Revealed by Ultra-deep Whole-Genome Sequencing. Nat. Neurosci. 24, 611. 10.1038/s41593-021-00830-8 PMC8533617 33753946

[B52] Saag L. Varul L. Scheib C. L. Stenderup J. Allentoft M. E. Saag L. (2017). Extensive Farming in Estonia Started through a Sex-Biased Migration from the Steppe. Curr. Biol. 27, 2185–2193. 10.1016/j.cub.2017.06.022 28712569

[B53] Saint Onge J. M. Brooks J. V. (2020). The Exchange and Use of Cultural and Social Capital Among Community Health Workers in the United States. Sociol. Health Illn 43, 299–315. 10.1111/1467-9566.13219 33211336

[B54] Saisho D. Purugganan M. D. (2007). Molecular Phylogeography of Domesticated Barley Traces Expansion of Agriculture in the Old World. Genetics 177, 1765–1776. 10.1534/genetics.107.079491 17947416 PMC2147987

[B55] Sanchez-Burks J. Lee F. Choi I. Nisbett R. Zhao S. Koo J. (2003). Conversing across Cultures: East-West Communication Styles in Work and Nonwork Contexts. J. Personal. Soc. Psychol. 85, 363–372. 10.1037/0022-3514.85.2.363 12916576

[B56] Stoneking M. Delfin F. (2010). The Human Genetic History of East Asia: Weaving a Complex Tapestry. Curr. Biol. 20, R188–R193. 10.1016/j.cub.2009.11.052 20178766

[B57] Stoof-Leichsenring K. R. Liu S. Jia W. Li K. Pestryakova L. A. Mischke S. (2020). Plant Diversity in Sedimentary DNA Obtained from High-Latitude (Siberia) and High-Elevation Lakes (China). Biodivers Data J. 8, e57089. 10.3897/BDJ.8.e57089 33364896 PMC7752886

[B58] Su B. Xiao J. Underhill P. Deka R. Zhang W. Akey J. (1999). Y-chromosome Evidence for a Northward Migration of Modern Humans into Eastern Asia during the Last Ice Age. Am. J. Hum. Genet. 65, 1718–1724. 10.1086/302680 10577926 PMC1288383

[B59] Sun N. Ma P.-C. Yan S. Wen S.-Q. Sun C. Du P.-X. (2019). Phylogeography of Y-Chromosome Haplogroup Q1a1a-M120, a Paternal Lineage Connecting Populations in Siberia and East Asia. Ann. Hum. Biol. 46, 261–266. 10.1080/03014460.2019.1632930 31208219

[B60] Tangkanchanapas P. Haegeman A. Ruttink T. Höfte M. De Jonghe K. (2020). Whole-Genome Deep Sequencing Reveals Host-Driven In-Planta Evolution of Columnea Latent Viroid (CLVd) Quasi-Species Populations. Int. J. Mol. Sci. 21, 3262. 10.3390/ijms21093262 PMC7246631 32380694

[B61] Uesugi R. Jouraku A. Sukonthabhirom Na Pattalung S. Hinomoto N. Kuwazaki S. Kanamori H. (2021). Origin, Selection, and Spread of Diamide Insecticide Resistance Allele in Field Populations of Diamondback Moth in East and southeastAsia. Pest Manag. Sci. 77, 313–324. 10.1002/ps.6020 33411414

[B62] Wang C.-C. Huang Y. Yu X. e. Chen C. Jin L. Li H. (2016). Agriculture Driving Male Expansion in Neolithic Time. Sci. China Life Sci. 59, 643–646. 10.1007/s11427-016-5057-y 27132019

[B63] Wang C.-C. Yeh H.-Y. Popov A. N. Zhang H.-Q. Matsumura H. Sirak K. (2021a). Genomic Insights into the Formation of Human Populations in East Asia. Nature 591, 413–419. 10.1038/s41586-021-03336-2 33618348 PMC7993749

[B64] Wang T. Wang W. Xie G. Li Z. Fan X. Yang Q. (2021b). Human Population History at the Crossroads of East and Southeast Asia since 11,000 Years Ago. Cell 184, 3829–3841. 10.1016/j.cell.2021.05.018 34171307

[B65] Wen B. Li H. Lu D. Song X. Zhang F. He Y. (2004). Genetic Evidence Supports Demic Diffusion of Han Culture. Nature 431, 302–305. 10.1038/nature02878 15372031

[B80] Wen S. Q. Yao H. B. Du P. X. Wei L. H. Tong X. Z. Wang L. X. (2019). Molecular Genealogy of Tusi Lu’s Family Reveals Their Paternal Relationship With Jochi, Genghis Khan’s Eldest Son. J. Hum. Genet. 64, 815–820. 10.1038/s10038-019-0618-0 31164702

[B66] Xiaowei M. Hucai Z. Shiyu Q. Yichen L. Fengqin C. Ping X. (2021). The Deep Population History of Northern East Asia from the Late Pleistocene to the Holocene. Cell 184, 3256–3266. 10.1016/j.cell.2021.04.040 34048699

[B67] Xu G. (2008). Building a Platform for East-West Communication in Stroke Research: Report of the Third International Stroke Summit, Wuhan, China, November 1-3, 2007. Cerebrovasc. Dis. 25, 279–280. 10.1159/000119636 18332622

[B68] Xu S. Jin L. (2008). A Genome-wide Analysis of Admixture in Uyghurs and a High-Density Admixture Map for Disease-Gene Discovery. Am. J. Hum. Genet. 83, 322–336. 10.1016/j.ajhg.2008.08.001 18760393 PMC2556439

[B69] Yang M. A. Fan X. Sun B. Chen C. Lang J. Ko Y.-C. (2020). Ancient DNA Indicates Human Population Shifts and Admixture in Northern and Southern China. Science 369, 282–288. 10.1126/science.aba0909 32409524

[B81] Yao H. B. Tang S. Yao X. Yeh H. Y. Zhang W. Xie Z. (2017). The Genetic Admixture in Tibetan-Yi Corridor. Am. J. Phys. Anthropol. 164, 522–532. 10.1002/ajpa.23291 28782792

[B82] Yao H. B. Wang C. C. Tao X. Shang L. Wen S. Q. Zhu B. (2016). Genetic Evidence for an East Asian Origin of Chinese Muslim Populations Dongxiang and Hui. Sci. Rep. 6, 38656. 10.1038/srep38656 27924949 PMC5141421

[B70] Yao H. Wang M. Zou X. Li Y. Yang X. Li A. (2021). New Insights into the fine-scale History of Western-Eastern Admixture of the Northwestern Chinese Population in the Hexi Corridor via Genome-wide Genetic Legacy. Mol. Genet. Genomics 296, 631–651. 10.1007/s00438-021-01767-0 33650010

[B71] Zhang D. Xia H. Chen F. Li B. Slon V. Cheng T. (2020). Denisovan DNA in Late Pleistocene Sediments from Baishiya Karst Cave on the Tibetan Plateau. Science 370, 584–587. 10.1126/science.abb6320 33122381

[B72] Zhao J. WurigemuleSun J. Sun J. Xia Z. He G. Yang X. (2020). Genetic Substructure and Admixture of Mongolians and Kazakhs Inferred from Genome-wide Array Genotyping. Ann. Hum. Biol. 47, 620–628. 10.1080/03014460.2020.1837952 33059477

[B73] Zhou X. Yu J. Spengler R. N. Shen H. Zhao K. Ge J. (2020). 5,200-year-old Cereal Grains from the Eastern Altai Mountains Redate the Trans-eurasian Crop Exchange. Nat. Plants 6, 78–87. 10.1038/s41477-019-0581-y 32055044

[B74] Zohary D. Hopf M. (1973). Domestication of Pulses in the Old World. Science 182, 887–894. 10.1126/science.182.4115.887 17737521

